# Supplementary figures and images for: Expression of the neurotrophic tyrosine kinase receptors, ntrk1 and ntrk2a, precedes expression of other ntrk genes in embryonic zebrafish
Source: PeerJ. 2020 Dec 22;8:e10479. doi: 10.7717/peerj.10479 (PMC7761192; doi:10.7717/peerj.10479)

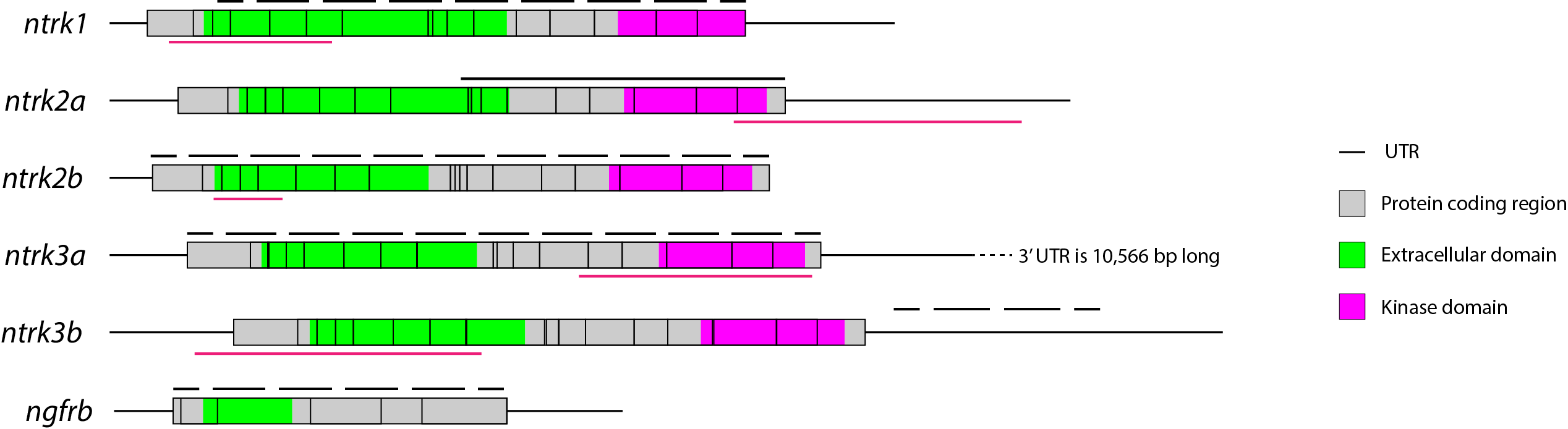

Supplement: Supplemental Information 1 — Gene diagrams show 5’ and 3’ UTRs, exon-exon junctions, protein coding regions, kinase domains, and extracellular domains. On top of each gene diagram, the black line represents the region spanned by our probe. Dashed lines were hydrolyzed and solid lines were not hydrolyzed. Below each gene diagram, the red line represents the region spanned by the probe used by Nittoli et al., 2018. Information from each gene was based on the longest isoform available in Zebrafish Ensembl release 92, and diagrams were each constructed using the same scale. Protein domains were assigned using the PRINTS database. [file peerj-08-10479-s001.png]

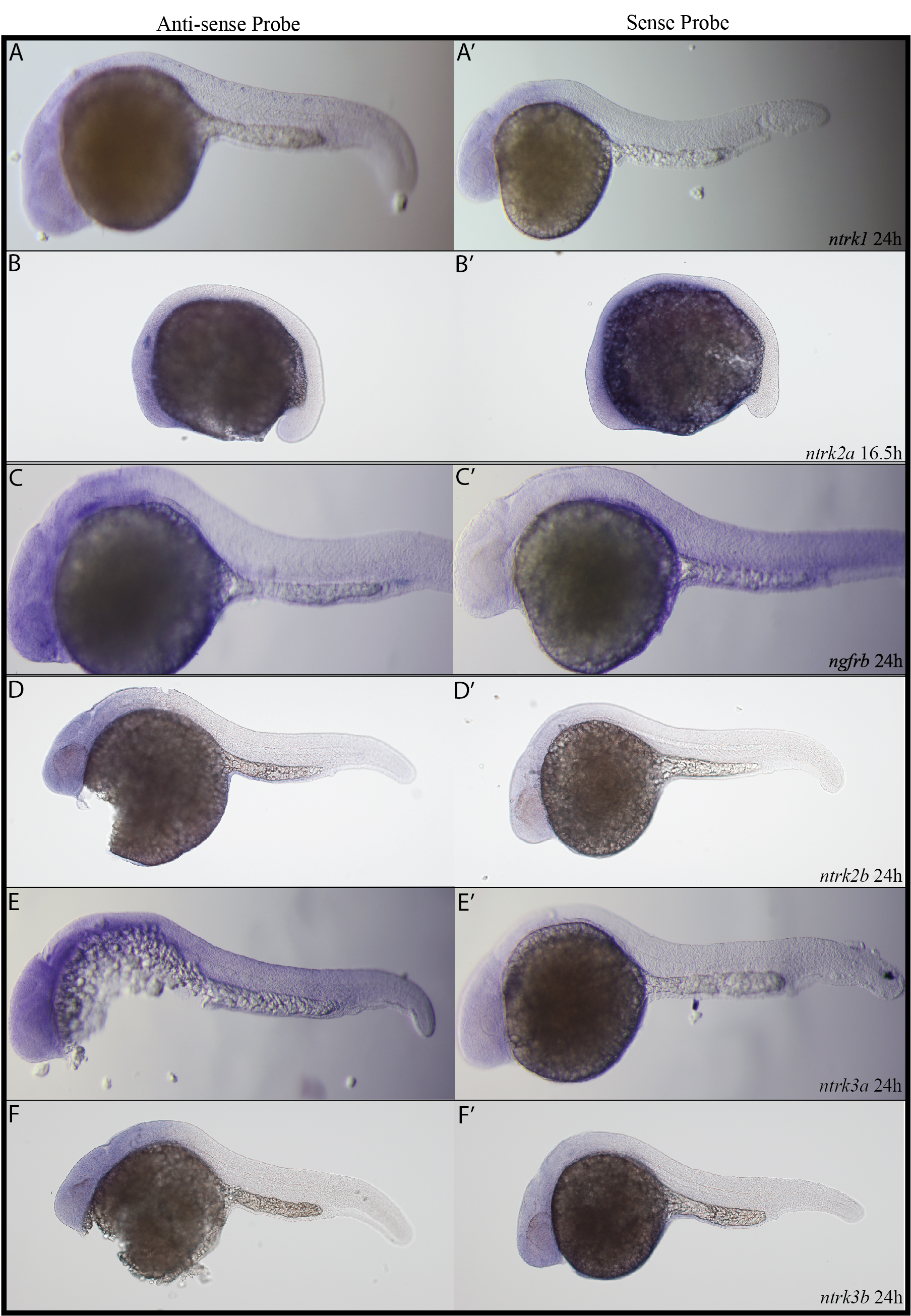

Supplement: Supplemental Information 2 — (A – F) Sense probes and (A’ – F’) anti-sense probes were used for in situ hybridizations. At 24 hpf, (A) the ntrk1 anti-sense probe resulted in staining in the spinal cord and (A’) the ntrk1 sense probe did not. At 16.5 hpf, (B) the ntrk2a anti-sense probe resulted in staining in cranial ganglia and (B’) the ntrk2a sense probe did not. At 24 hpf, (C) the ngfrb anti-sense probe resulted in staining in cranial ganglia and (C’) the ngfrb sense probe did not. At 24 hpf, (D) the ntrk2b anti-sense probe resulted in staining in the forebrain and (D’) the ntrk2b sense probe did not. At 24 hpf, (E) the ntrk3a anti-sense probe resulted in staining in the forebrain and (E’) the ntrk3a sense probe did not. At 24 hpf, (F) the ntrk3b anti-sense probe resulted in staining in the forebrain, midbrain and hindbrain, while (F’) the ntrk3b sense probe did not. We think it worth noting that we noticed the most variability with our ntrk3b probe. Within a round of in situ hybridizations we saw similar staining from embryo to embryo, but from round to round, the staining could be more intense (as in Figure 6) or less intense (as in F’). We speculate that the variability could be because our ntrk3b probe recognizes the 3’ UTR (see Supplemental Figure 1). [file peerj-08-10479-s002.jpg]
